# Measuring group synchrony: a cluster-phase method for analyzing multivariate movement time-series

**DOI:** 10.3389/fphys.2012.00405

**Published:** 2012-10-19

**Authors:** Michael J. Richardson, Randi L. Garcia, Till D. Frank, Madison Gergor, Kerry L. Marsh

**Affiliations:** ^1^Department of Psychology, Center for Cognition, Action, and Perception, University of CincinnatiCincinnati, OH, USA; ^2^Department of Psychology, University of ConnecticutStorrs, CT, USA; ^3^Center for the Ecological Study of Perception-Action, University of Connecticut, StorrsCT, USA; ^4^Department of Psychology, Colby CollegeWaterville, ME, USA

**Keywords:** cluster phase method, group synchrony, interpersonal coordination, group processes, multivariate analysis

## Abstract

A new method for assessing group synchrony is introduced as being potentially useful for objectively determining degree of group cohesiveness or entitativity. The cluster-phase method of Frank and Richardson (2010) was used to analyze movement data from the rocking chair movements of six-member groups who rocked their chairs while seated in a circle facing the center. In some trials group members had no information about others' movements (their eyes were shut) or they had their eyes open and gazed at a marker in the center of the group. As predicted, the group level synchrony measure was able to distinguish between situations where synchrony would have been possible and situations where it would be impossible. Moreover, other aspects of the analysis illustrated how the cluster phase measures can be used to determine the type of patterning of group synchrony, and, when integrated with multi-level modeling, can be used to examine individual-level differences in synchrony and dyadic level synchrony as well.

A common feature of many social activities, including a group of friends walking to class together, an audience swaying to the music of their favorite rock band at a concert, or a highly trained rowing team racing down a river, is the synchrony or coordination that occurs between the movements of the actors involved. Although the magnitude or stability of movement synchrony can differ across different social situations, it is a natural part of interpersonal behavior (Bernieri and Rosenthal, 1991; Fowler et al., 2008; Marsh et al., 2009; Miles et al., 2009; Richardson et al., 2010) and can result both intentionally due to intrinsic task requirements (i.e., when rowing) and spontaneously (i.e., when friends are walking to class) due to the myriad of perceptual-motor couplings that exist during social interaction (Schmidt et al., 1998; Repp and Penel, 2004; Schmidt and Richardson, 2008).

Movement synchrony may be a fundamental means of becoming a social unit with others (Marsh et al., 2009; Marsh, 2010), and of blending the boundaries of one's self with another (Paladino et al., 2010). Synchrony helps build rapport with others (Bernieri et al., 1996; Chartrand and Bargh, 1999; Hove and Risen, 2009; Marsh et al., 2009), and telegraphs to outsiders that individuals are a social unit and have rapport (Macrae et al., 2008; Lakens, 2010; Lakens and Stel, 2011). Being psychologically distanced from another individual can cause a reduction in interpersonal synchrony (Miles et al., 2010). Synchrony not only facilitates memory for those we synchronize with (Miles et al., 2010) but can more generally facilitate performance of cognitive or linguistic tasks (Richardson et al., 2005; Shockley et al., 2009). Thus, developing a detailed understanding of why and when it does or does not occur has significant implications for understanding social behavior.

It should come as no surprise then that there is a large body of research that has attempted to examine and model such behavior (see Schmidt and Richardson, 2008; Marsh et al., 2009, for reviews). Despite the fact that movement synchrony presumably can occur between 3 or more individuals, research on between-person movement synchronization has, with rare exception, been limited to the movement coordination of dyads. Typically, these studies involve recording the movements of a single limb from each participant (e.g., each participant's leg, arm, or hand movements) under different intentional and social constraints (e.g., Boker and Rotondo, 2003). Movement synchrony or coordination is then quantified using various bivariate measures, such as relative phase, frequency difference, frequency, or cross-spectral coherence, cross-correlation, and cross-recurrence analysis. Interaction between two subsystems, in general, and phase synchronization between two dynamical systems, in particular, can also be quantified by means of various entropy measures (Tass et al., 1998; Wojcik et al., 2001), mutual information (Palus, 1997), phase distribution (Frank et al., 2000), and phase diffusion index measures (e.g., Pikovsky et al., 2001; Schelter et al., 2007). The most commonly used quantifications are the mean and SD of the relative phase time-series ($\bar{\phi }$ and *SD*ϕ, respectively) that occurs between the movements of the two participants, where the relative phase time-series, ϕ(t), is calculated as the difference between the phase angles, θ (t), of the two movement time-series [i.e., θ_2_(*t*)− θ_1_(*t*)]. For 1-to-1 frequency locked synchrony, $\bar{\phi }$ is used to identify the degree to which the pattern of coordination is equal to or shifted away from one of the two stable states of interlimb coordination, namely $\bar{\phi }$ = 0° and 180° (referred to as inphase and antiphase coordination, respectively). *SD*ϕ is used to determined the stability of the coordination, with greater values of *SD*ϕ corresponding to weaker or less stable states of coordination (e.g., Schmidt et al., 1990; Richardson et al., 2007). The distribution of ϕ(t) has also been used to quantify the degree of spontaneous synchrony, in that observing a greater number of relative phase angles around 0° and 180° is indicative of intermittent or relative coordination (Schmidt and O'Brien, 1997; Richardson et al., 2007).

What about the movement synchrony or coordination that involves more than two people? There are important theoretical reasons why studying synchrony at a group level may be important. Whereas dyadic interpersonal coordination forms the basis for joint action (Clark, 1996), communication, rapport, and the formation of relationships (Tickle-Degnen and Rosenthal, 1990; Fiske, 1992), group-level synchrony may be an important behavioral indicator of group cohesiveness, the degree to which a group has a sense of “groupness,” or existence as an *entity* (i.e., group entitativity, a term coined by Campbell, 1958). Group cohesiveness, entitativity, and social identification are viewed as crucial processes in understanding a range of phenomena, from dysfunctional group decision-making (Janis, 1982), to social influence (Festinger et al., 1950), intergroup conflict, and social identity processes (Simon and Pettigrew, 1990; Tsui and Gutek, 1999; Tajfel and Turner, 2004). To date, however, nearly all means of assessing degree of groupness involves self-report (Lickel et al., 2000). We hypothesize that, as occurs with dyadic interpersonal synchrony (Miles et al., 2010), group synchrony may occur when individuals have mutual interpersonal connection with others. These may be due to valence bonds (friendship and liking) or due to some functional reasons for their connection (belonging to a family, or a workgroup that must cooperate). Thus, a behavioral means of assessing group synchrony could potentially revolutionize the study of group processes. Furthermore, if group synchrony measures are integrated with methodological techniques that allow for multi-level modeling of data it would provide the ability to look at both dyadic and individual level synchrony within a group, as well as group-level differences in synchrony, that is, the ability to empirically determine the level at which synchrony is occurring (Bond and Kenny, 2002). For example, being able to identify individual differences in synchrony with the group provides the potential to understand how some individuals within a group may be strongly pulled to coordinate with others, whereas others may tend to be relatively impervious to such social influence.

Almost no research studies have examined the movement synchronization that occurs between 3 or more individuals. One exception is work by Néda et al. (2000a,b) in which they examined the synchronized clapping of an audience in a naturalistic setting. As this latter work points out, group process research requires that researchers examine not only consequent emergent synchrony as a final product, but also the individual level movements that contribute to synchrony or group coordination. Being able to assess the movements of each individual in the process of examining group synchrony is therefore critical. One reason for the lack of such research concerns the inability of researchers to simultaneously record the limb and body movements of multiple individuals. However, recent technical advances in multi-sensor motion tracking systems (e.g., NDI's optical tracking systems, Polhemus's Liberty, or Latus magnetic tracking systems) that can provide time series records of the limb or body movements of many individuals means that this is no longer a barrier.

A second reason for the limited group synchrony research is a lack of verified statistics for quantifying the magnitude and stability of the synchrony that can occur between multiple movement time-series. This latter issue is really a two-fold issue. First, how can one effectively measure the overall synchrony of a group of individuals as a whole? Second, how can one effectively measure the degree to which the movements of any one individual in the group are synchronized to the movements of a group as a whole? Here, we address these questions by adapting and testing a *cluster phase* method recently proposed by Frank and Richardson (2010). The method is based on the Kuramoto order parameter^1^ (Kuramoto, 1984, 1989), which has been used previously to examine the phase synchronization of many-body systems (e.g., a large set of oscillators), such as the synchronized firefly flashing and chirping of crickets, (see Strogatz, 2000, for a review), and synchronized applause (Néda et al., 2000a,b). The method directly quantifies phase synchronization in noisy experimental multivariate data.

## Cluster phase quantification of group synchrony

The Kuramoto based cluster phase method proposed by Frank and Richardson (2010) can be used to quantify phase synchronization in noisy experimental multivariate data as follows.

First, for *n* movement (participant) times-series, *x*_1_(*t*_*i*_),…, x_*k*_(*t*_i_), where *k* = 1,…, *n* and *i* = 1,…, *T* time steps, calculate the phase times-series for each movement, θ_*k*_, in radians [–π π]. This can be done either using the Hilbert transform or a frequency normalized continuous phase calculation (see Pikovsky et al. (2001) for an overview of these standard phase calculation methods).

Second, calculate the group phase time-series or *cluster* phase *q*(*t*_*i*_) from:

$$\overset{´}{q}(t_{i})=\frac{1}{n}\sum_{k\;=\;1}^{n}\text{exp}(i\theta_{k}(t_{i}))$$

and

$$q(t_{i})=\text{atan2}(\overset{´}{q}(t_{i}))$$

where *n* = the number of movements, $i=\sqrt{-1}$ (when not used as a time step index), and $\overset{´}{q}(t_{i})$ and *q*(*t*_*i*_) are the resulting cluster phase in complex and radian [–ππ] form, respectively.

Third, calculate the relative phases for the individual movements with respect to the cluster phase as:

$$\phi_{k}(t_{i})=\theta_{k}(t_{i})-q(t_{i})$$

where *k* = 1 ,…, *n* and ϕ_k_ is the relative phase times for each movement (participant).

Forth, compute the mean relative phase ${\bar{\phi }}_{k}$ and the degree of synchrony ρ_*k*_ for every movement *k* with respect to the group behavior *q* from:

$$\begin{array}{c} {\bar{\overset{´}{\phi }}}_{k}=\frac{1}{N}\sum_{i\;=\;1}^{N}\text{exp}(i\phi_{k}(t_{i}))\\ {\bar{\phi }}_{k}=\text{atan2}({\bar{\overset{´}{\phi }}}_{k}), \end{array}$$

and

$$\rho_{k}=\left|{\bar{\overset{´}{\phi }}}_{k}\right|$$

where *N* is the number of time steps ${\bar{\overset{´}{\phi }}}_{k}$ and ${\bar{\phi }}_{k}$ is the mean cluster phase in complex and radian [–π π] form, and ρ_*k*_∈ [0, 1]. Here, ρ_k_ corresponds to the inverse of the circular variance^2^ of ϕ_*k*_(*t*_*i*_). Thus, if ρ_*k*_ = 1 the movement is in complete synchronization with the group (i.e., the phase of the movement at any time step *t*_*i*_ is equivalent to the group phase shifted by a constant phase). If ρ_*k*_ = 0 the movement is completely unsynchronized to the group. Note that ${\bar{\phi }}_{k}$ captures the phase shift of a movement with respect to the group behavior *q*. For stable synchrony (i.e., ρ_*k*_ tending toward 1) it can be used to compare if movements have the same mean phase with the group and, thus, determine the between movement relative phase relations. For instance, if ${\bar{\phi }}_{n}={\bar{\phi }}_{m}$ then the mean relative phase between movement (participant) *m* and *n* is zero and they are perfectly inphase with one another.

Finally, the degree of synchronization of the group as a whole ρ_group_at every time step *t*_i_ is defined by:

$$\rho_{\text{group},i}=\left|\frac{1}{n}\sum_{k\;=\;1}^{n}\text{exp}\{i(\phi_{k}(t_{i})-{\bar{\phi }}_{k})\}\right|$$

where ρ_group,*i*_∈ [0, 1] and the mean degree to group synchronization is computed as:

$$\rho_{\text{group}}=\frac{1}{N}\sum_{i\;=\;1}^{N}\rho_{\text{group},i}$$

As with ρ_*k*_ above, the larger the value of ρ_group,*i*_ and ρ_group_ (i.e., the closer to 1) the larger the degree of group synchronization. Note that ρ_group_ provides a single measure of group synchrony for a behavioral period (trial), whereas ρ_group,i_ provides a continuous measure of group synchrony^3^.

## Experimental test of method

To test the effectiveness of the above method, we conducted a study of group synchrony in which groups of six participants, arranged in a circle, rocked in rocking chairs at a self-selected or predetermined frequency. This social coordination paradigm was chosen for two reasons. First, the rocking chair movements of many participants could be recorded easily by placing motion tracking sensors unobtrusively behind the head rest of each participant's chair. Second, previous research has found that the natural period of rocking chairs is quite stable such that the individual differences in participant weight has a negligible effect on movement frequency (Richardson et al., 2007). Third, previous research (Richardson et al., 2007) has demonstrated that rocking chair movements can be synchronized, but only when participants have information about their co-participant's movements (e.g., can see each other). In short, this methodology provided a way to examine the effectiveness of the cluster phase statistics in determining the phase synchronization of multivariate time-series movement data under intentional (eyes-open) and chance (eyes-closed) levels of coordination.

## Method

### Participants

Eight groups of six participants (48 participants in mixed gender groups) were recruited for the study. All participants were Colby college undergraduate students who completed the experiment for partial course credit or monetary incentive (US $6.00). All participants were naïve to the study's purpose and had not previously participated in a study on rhythmic or social movement coordination^4^.

### Materials

Participants sat and rocked in six identical wooden rocking chairs. The chairs were positioned evenly around a central 10 × 10 cm target that stood on a 5 cm wide by 1.2 m high stand. The chairs formed a circle with a radius of 1.25 m, with the radius assessed from the center of target to the front of the chairs. The Euclidean *x*-*y*-*z* movements of each rocking chair was recorded at 120 Hz using a magnetic tracking system (Polhemus Liberty, Polhemus Corporation, Colchester, VT), with the motion sensors attached unobtrusively to the back of each chairs' headrest.

### Procedure

Upon arrival each participant was randomly assigned to one of the six chairs. Participants were instructed to rock at a self-selected frequency (*groups 101–104*) or at a frequency of 0.6 Hz^5^ (*groups 201–204*). With respect to the latter groups, a metronome beat was presented to participants for 30 s prior to beginning the experiment trials so that participants could practice rocking at this frequency. The metronome was *not* presented during the experimental trials. This metronome condition was employed to ensure that participants rocked at a tempo more consistent with the natural rocking tempo of the chairs, as deviations from a systems natural movement frequency can decrease coordination stability (e.g., Richardson et al., 2007; Schmidt and Richardson, 2008).

Every group completed three 3 min trials in the following order: one *eyes closed* trial; and two *eyes open* trials. For the eyes closed trial, participants were instructed to either rock at their own self-selected tempo or at the practice (0.6 Hz) frequency (depending on group) with their eyes closed. This trial allowed for a measure of chance level synchrony as participants had no visual information about their co-participants' movements. For the eyes open trials, participants were instructed to rock at a self-selected frequency or at the practice frequency (depending on group) while attempting to synchronize their rocking chair movements as a group. To control for looking direction, all participants were instructed to look at the central target. No instructions as to the form or pattern of synchrony were provided.

### Data reduction and signal processing

**Figure 1:**
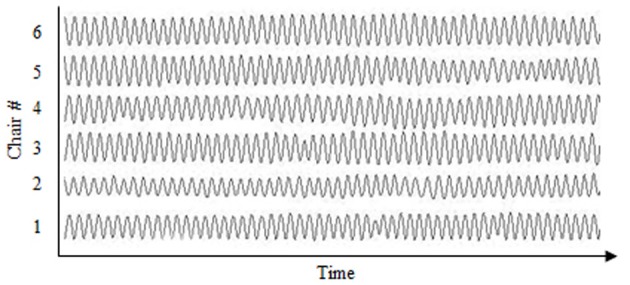
**Example time-series of the rocking chairs' z-direction (up-down) movements over time**.

Due to the circular arrangement of the chairs the z-direction (up-down) of movement was extracted from the movement recordings for analysis as it was the only uniform direction (motion time-series) across chairs (see Figure 1). Prior to performing the analysis, the movement time-series were down-sampled from 120 to 60 Hz, centered around zero, and low-pass filtered using a 10 Hz Butterworth filter. The Hilbert transform was employed to calculate the phase times-series for each movement. In addition to performing the cluster phase analysis defined above, a peak-picking algorithm was used to obtain the mean frequency (Hz) of the chair movements for each trial and was calculated as the inverse of the mean time between the points of maximum extension. This frequency analysis revealed that participants produced the equivalent movement frequencies in both the self-paced (*M* = 0.58, *SD* = 0.06) and metronome paced (*M* = 0.60, *SD* = 0.04) conditions and for both the eyes-closed (*M* = 0.59, *SD* = 0.08) and eyes-open (*M* = 0.50, *SD* = 0.03) conditions. This is consistent with previous rocking chair research (Richardson et al., 2007), and reflects the tendency of participants to produced movements close to a systems natural or resonant frequency (Kugler and Turvey, 1987; Richardson et al., 2007). Given that an initial analysis of the data revealed no significant effects of whether individuals' pacing was set by the experimenter or not, this factor was removed from the analysis and the eight groups were treated as equivalent with respect to pacing.

## Results and discussion

The aim of the current experiment was to demonstrate that the cluster phase method proposed by Frank and Richardson (2010) could be used to effectively measure group synchrony. As a way of illustrating the effectiveness of the differing cluster phase statistics we first present a qualitative assessment of ρ_group,*i*_ (which provides a continuous measure of group synchrony) and the cluster phase calculations of mean and SD of relative phase (which can be used to illustrate the patterning of the synchrony). Following this, we then present a quantitative (statistical) analysis of the cluster phase statistics ρ_group_, ρ_*k*_, and ρ_*d*_ (see below for definitions) to objectively determine the effectiveness with which they can be used to (1) measure the presence and magnitude of group movement synchrony as a whole, (2) the degree to which the different individuals in the group were synchronized to the movements of the group as a whole, respectively, and (3) the degree to which two individuals within the group are synchronized with each other.

Recall that the eyes-closed condition enabled a measure of chance level coordination, that is, a statistical magnitude by which actual coordination could be assessed against. Thus, while the overall magnitude of the cluster phase statistics, ρ_group,*i*_, ρ_group_, and ρ_*k*_ (i.e., the closer to 1) is indicative of greater synchrony, the instructive comparison for both the qualitative and quantitative analysis presented below is the magnitude difference between the eyes-closed and eyes-open conditions. Specifically, the magnitude of the cluster phase statistics, ρ_group,*i*_, ρ_group_, and ρ_*k*_ should be greater for the eyes-open condition compared to the eyes-closed condition.

### Qualitative analysis

**Figure 2:**
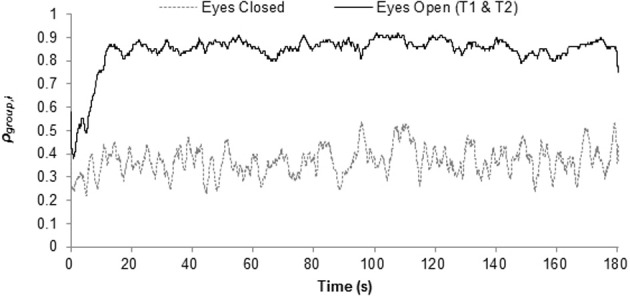
**Continuous group synchrony, ρ_**group**,*i*_ averaged across group and trial**.

An inspection of Figure 2, which plots ρ_group,*i*_ averaged across group as a function of time, provides preliminary support for the cluster phase method. Specifically, the data presented in Figure 2 reveals how following an initial transient period of approximately 15 s, ρ_group,*i*_ for both of the eyes-open trials remained at a much greater level across the course of the trials than that observed for the eyes-closed condition. A similar pattern was exhibited for each of the eight groups, with ρ_group,*i*_ ranging between approximately 0.7 and 0.9 for the coordination (eyes-open) condition and between approximately 0.2 and 0.4 for the chance level (eyes-closed) condition.

With respect to the patterning of the synchrony that emerged during the intentional coordination (eyes-open) trials, individuals within a group tended to exhibit an inphase pattern of coordination to the group as a whole (as measured by ${\bar{\phi }}_{\text{clus}.}$, *M* = 0.38, *SD* = 15.21), with a modest degree of stability (as measured by SDϕ_clus._, *M* = 41.34, *SD* = 20.94). The individual measures, by chair number, of ${\bar{\phi }}_{\text{clus}.}$ and SDϕ_clus._ are displayed in Table 1. Note that although ${\bar{\phi }}_{\text{clus}.}$ and SDϕ_clus._ could be determined for the eyes-closed condition the circular nature of relative phase means that such calculations are trivial for chance (or intermittent) coordination and do not reflect a meaningful synchrony relationship.

**Table 1:** **Mean (SD) cluster relative phase (${\bar{\phi }}_{\text{clus}.}$ and *SD*ϕ_**clus.**_, respectively)**.

**Group no.**	**Trial**	**Chair number**					
		**1**	**2**	**3**	**4**	**5**	**6**
101	Eyes Closed	−	−	−	−	−	−
	Eyes Open (T1)	−12.4 (17.6)	−3.9 (23)	3.4 (13.6)	5.2 (24)	−4.5 (49.3)	10.5 (12.1)
	Eyes Open (T2)	−9.9 (18.3)	1.2 (21)	−2.3 (17.5)	4.5 (27.1)	−6.2 (23.3)	12.7 (15.8)
102	Eyes Closed	−	−	−	−	−	−
	Eyes Open (T1)	−3.6 (30)	−10.8 (28.6)	−0.9 (29.2)	0.8 (20.4)	56 (69.5)	−0.1 (20.1)
	Eyes Open (T2)	6.3 (35.1)	−21.7 (22.5)	15.2 (27.4)	7 (19.6)	−4.7 (32.4)	−1.7 (28)
103	Eyes Closed	−	−	−	−	−	−
	Eyes Open (T1)	−0.5 (18.5)	−4.2 (22.9)	7.5 (19)	13.2 (28.3)	2.6 (46.6)	−48.4 (64.2)
	Eyes Open (T2)	−3.4 (22.1)	−5.9 (23.8)	0.8 (21)	−2.5 (28.9)	0.7 (44.9)	39.2 (69.7)
104	Eyes Closed	−	−	−	−	−	−
	Eyes Open (T1)	−1.8 (17)	31.1 (37.9)	−5.9 (19.7)	−21.4 (46)	11.9 (24)	−13.7 (23.3)
	Eyes Open (T2)	10 (32.4)	27.1 (30.6)	−19.7 (36)	−14.2 (32.5)	5.6 (28)	−9 (21.9)
201	Eyes Closed	−	−	−	−	−	−
	Eyes Open (T1)	−15.3 (13.2)	4.8 (30.2)	15.3 (17.5)	10.7 (16.5)	−11.7 (24.2)	−3.5 (16.2)
	Eyes Open (T2)	−21.3 (17.1)	3.1 (45)	4.8 (18.9)	4.2 (17.5)	16.6 (25)	−5.8 (15.1)
202	Eyes Closed	−	−	−	−	−	−
	Eyes Open (T1)	11.1 (24.7)	−0.8 (23.4)	−12 (54.1)	−20.5 (30.3)	14.4 (36.7)	3.9 (34)
	Eyes Open (T2)	0.3 (27.2)	7.1 (22.4)	−8.3 (62)	−13.8 (43.5)	19.4 (26.3)	−30.3 (64.3)
203	Eyes Closed	−	−	−	−	−	−
	Eyes Open (T1)	13 (43.6)	−8.9 (21.8)	−8.1 (21.2)	−9 (23.8)	8.8 (24.2)	8.1 (32.7)
	Eyes Open (T2)	12.2 (62)	−4.4 (31.4)	12.8 (24)	−1 (25.5)	6.8 (50.3)	−32.9 (56.3)
204	Eyes Closed	−	−	−	−	−	−
	Eyes Open (T1)	−30.3 (33.5)	−4.8 (23.7)	6 (33.3)	13.6 (26.4)	7.9 (30.3)	5.3 (31)
	Eyes Open (T2)	−18.7 (24)	−0.9 (23)	2.7 (24)	−9.2 (24.7)	20.7 (32.4)	6.9 (23.3)

### Quantitative analysis

**Figure 3:**
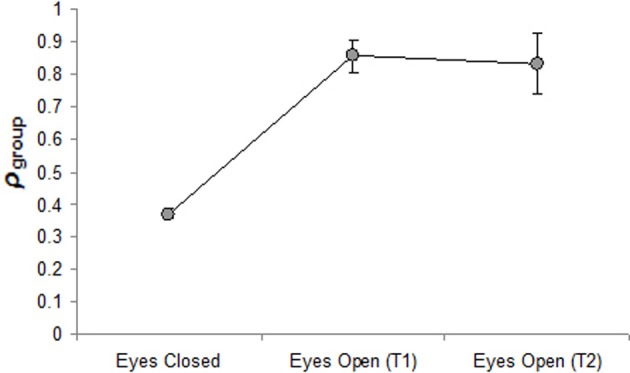
**Mean group synchrony, ρ_**group**_, as a function of condition and trial (T)**.

The ρ_group_ and ρ_*k*_ measures for the eyes-closed and both eyes-open trials are displayed in Table 2. To assess the validity of the ρ_group_ statistic in measuring group based synchrony, a One-Way repeated measures ANOVA was conducted to compare the three trials (*eyes closed*, *eyes-open trial one*, and *eyes-open trial two*). If ρ_group_ is a valid measure of group synchrony then it should be a significantly greater (closer to 1) in the eyes-open conditions than in the eyes-closed condition, where any synchrony that occurs is simply due to chance. This was indeed the case, with the omnibus ANOVA revealing a significant effect of trial, *F*_(2, 14)_ = 189.10, *p* < 0.01, η^2^ = 0.96 (see Figure 3) and a *post-hoc* analysis (Tukey-HSD) finding that the two eyes-open trials were significant greater than the eyes-closed condition (both *p* < 0.01), but were not significantly different from each other (*p* > 0.95).

**Table 2:** **Individual (ρ_k_) and group (ρ_**group**_) cluster amplitudes**.

**Group no.**	**Trial**	**Chair number**						
		**1**	**2**	**3**	**4**	**5**	**6**	ρ_**group**_
101	Eyes Closed	0.30	0.27	0.42	0.40	0.38	0.31	0.36
	Eyes Open (T1)	0.95	0.92	0.97	0.91	0.63	0.98	0.89
	Eyes Open (T2)	0.95	0.93	0.95	0.89	0.92	0.96	0.93
102	Eyes Closed	0.31	0.42	0.14	0.32	0.34	0.46	0.36
	Eyes Open (T1)	0.86	0.88	0.87	0.94	0.26	0.94	0.80
	Eyes Open (T2)	0.81	0.92	0.89	0.94	0.84	0.88	0.88
103	Eyes Closed	0.36	0.34	0.30	0.35	0.34	0.40	0.36
	Eyes Open (T1)	0.95	0.92	0.95	0.88	0.67	0.37	0.80
	Eyes Open (T2)	0.93	0.91	0.93	0.87	0.69	0.26	0.77
104	Eyes Closed	0.36	0.35	0.41	0.39	0.38	0.36	0.38
	Eyes Open (T1)	0.96	0.78	0.94	0.68	0.91	0.92	0.87
	Eyes Open (T2)	0.84	0.86	0.80	0.84	0.88	0.93	0.86
201	Eyes Closed	0.33	0.38	0.48	0.41	0.30	0.25	0.37
	Eyes Open (T1)	0.97	0.86	0.95	0.96	0.91	0.96	0.94
	Eyes Open (T2)	0.96	0.69	0.95	0.95	0.91	0.97	0.90
202	Eyes Closed	0.40	0.22	0.32	0.43	0.58	0.30	0.41
	Eyes Open (T1)	0.91	0.92	0.55	0.86	0.79	0.82	0.81
	Eyes Open (T2)	0.89	0.92	0.41	0.71	0.90	0.37	0.71
203	Eyes Closed	0.37	0.47	0.17	0.39	0.35	0.34	0.36
	Eyes Open (T1)	0.71	0.93	0.93	0.91	0.91	0.84	0.87
	Eyes Open (T2)	0.42	0.85	0.91	0.90	0.61	0.52	0.71
204	Eyes Closed	0.35	0.41	0.34	0.33	0.46	0.33	0.38
	Eyes Open (T1)	0.83	0.91	0.83	0.89	0.86	0.85	0.87
	Eyes Open (T2)	0.91	0.92	0.91	0.91	0.84	0.92	0.90

In addition to the group-based measure of synchrony, ρ_group_, Frank and Richardson (2010) also proposed that the cluster phase statistic of ρ_*k*_ could be used to measure the extent to which each individual is synchronizing with his or her group. Just as in the analyses using group based synchrony above, this would be validated by finding significantly more individual synchrony, greater magnitudes of ρ_*k*_ (closer to 1), in the eyes open compared to the eyes-closed condition. Of more import is the possibility that ρ_*k*_ can be used to examine the variation in the extent to which differing individuals within a group are synchronized with their group as a whole. This latter possibility was investigated using multilevel modeling with 2 levels (individual crossed with trial is level 1 and group is level 2). The variable Condition (fixed effect at level 1) was dummy coded with two indicator variables: an eyes closed indicator variable, as well as an eyes open trial 1 variable; thus, eyes open trial 2 was the comparison group. As in the group-based synchrony analyses above we found a significant difference in individual synchrony between the eyes closed condition and the eyes open trial 2 condition (*b* = −0.47, *p* < 0.01) with the eyes closed condition eliciting less individual synchrony. Furthermore, there was no statistically significant difference in individual synchrony between eyes open, trial 1 and eyes open, trial 2 (*b* = 0.02, *p* = 0.32).

Interestingly there was statistically significant individual variance in this synchrony in all three trials with the most individual synchrony in the eyes open conditions, as would be expected; however, it was not large (eyes closed: ${\hat{\sigma }}_{\rho_{k,\text{individual}}}^{2}=0.006$, *p* < 0.01; eyes open trial 1: ${\hat{\sigma }}_{\rho_{k,\text{individual}}}^{2}=0.02$, *p* < 0.01; eyes open trial 2: ${\hat{\sigma }}_{\rho_{k,\text{individual}}}^{2}=0.03$, *p* < 0.01). Variance in synchrony at the individual level measures whether or not some individuals are more synchronized with their groups than other individuals in each condition. There was also statistically significant covariance between individual synchrony in the first eyes open trial and in the second, ${\hat{\sigma }}_{\rho_{k,\text{individual}}}^{2}=0.55$, *p* < 0.01, which indicates that there is some consistency in individual synchrony across trials; that is, those who individually synchronize to their groups in the first eyes open trial tended to individually synchronize in the second eyes open trial. The covariances between the eyes open trials and the eyes closed trial were zero—as one would expect since the individuals who become synchronized in the eyes closed trials are random and not necessarily the ones who *tend* to synchronize. Likewise, we also tested if there was group based individual synchrony. This effect measures the extent to which some groups' *members* are more synchronized with the group than other groups' members. For example, some groups may have individuals that are strongly influenced by others (regardless of whether there is a group-level synchrony that is emerging) whereas others might not. Since there were no systematic differences in how groups were created we might not expect there to be any group-based variance. Indeed this is the case, there was no group variance—thus groups do not vary in their overall levels of individual synchrony.

### Dyadic synchrony

While the Frank and Richardson (2010) cluster phase method examined here enables a measure of group synchrony (ρ_group_) and the degree to which each individual is synchronizing with the group (ρ_*k*_), one should still examine dyadic synchrony (i.e., the synchrony between pairs of individuals in a group). That is, for each pair of phase time series θ_*k*_ and θ_*k*'_, with *k* ≠ *k*', one should examine the degree of dyadic synchronization, ρ_*d*_. This can be obtained by first calculating the relative phases for each pair of individuals within a group,

$$\phi_{d}(t_{i})=\theta_{k}(t_{i})-\theta_{k^{\prime }}(t_{i})$$

where *k* = 1 ,…, *n* and ϕ_*d*_ is the relative phase times series for each pair *d* = 1,…, *n*, and then by computing the degree of dyadic synchrony, ρ_*d*_ for every pair *d* from:

$${\bar{\overset{´}{\phi }}}_{d}=\frac{1}{N}\sum_{i\;=\;1}^{N}\text{exp}(i\phi_{d}(t_{i}))$$

and

$$\rho_{d}=\left|{\bar{\overset{´}{\phi }}}_{d}\right|$$

where *N* is the number of time steps ${\bar{\overset{´}{\phi }}}_{d}$ is the mean dyadic relative phase in complex form, and ρ_*d*_∈ [0, 1]. Again, ρ_*d*_ corresponds to the inverse of the circular variance of ϕ*d*(*t*_*i*_), where ρ_*d*_ = 0 reflects no synchrony and ρ_*d*_ = 1 reflects perfect dyadic synchrony.

See Table 3 for the ρ*d* values by dyad within each group. Multilevel modeling, with dyad as the unit of analysis (level 1) controlling for individual and group as level 2, was again used to test for variation in dyadic synchrony at the individual and group levels. A Social Relations Model (Kenny, 1994) approach to the decomposition of variance was used with constraints to account for the symmetric nature of the measurement (i.e., group member A's synchrony with group member B is equal to group member B's synchrony with group member A). For this analysis, only the eyes open trial two condition was used. Interestingly, there was statistically significant individual variance in dyadic synchrony, σ^2^_*d*,individual_ = 0.014, *p* < 0.01. That is, some individuals were more synchronized with the others in the group, on a pairwise basis. There was no group-based variance in dyadic synchrony however, σ^2^_*d*,group_ = 0.017, *p* = 0.13. Group-based variance here measures the extent to which some groups had pairs of members that were more synchronized with each other than were pairs in other groups. Even though group-based variance was not found in this context, in either the dyadic or individual measure of synchrony, there may be other contexts in which we would expect variation across groups to be present. For example, if some groups had pairs of friends in a group and other groups did not, then group-level differences in dyadic synchrony would be expected.

**Table 3:** **Dyadic, ρ_Dyad_, Cluster Amplitudes**.

**Group no.**	**Chair no.**	**Chair number**				
		**2**	**3**	**4**	**5**	**6**
101	1	0.90	0.88	0.85	0.83	0.89
	2	−	0.85	0.89	0.82	0.88
	3	−	−	0.84	0.91	0.95
	4	−	−	−	0.84	0.83
	5	−	−	−	−	0.90
102	1	0.70	0.70	0.76	0.69	0.86
	2	−	0.84	0.90	0.82	0.77
	3	−	−	0.91	0.68	0.78
	4	−	−	−	0.73	0.82
	5	−	−	−	−	0.78
103	1	0.87	0.86	0.81	0.63	0.19
	2	−	0.85	0.78	0.59	0.21
	3	−	−	0.82	0.61	0.21
	4	−	−	−	0.50	0.18
	5	−	−	−	−	0.14
104	1	0.70	0.61	0.62	0.82	0.73
	2	−	0.67	0.68	0.76	0.76
	3	−	−	0.66	0.68	0.70
	4	−	−	−	0.67	0.82
	5	−	−	−	−	0.80
201	1	0.62	0.91	0.91	0.82	0.93
	2	−	0.59	0.60	0.63	0.67
	3	−	−	0.91	0.85	0.90
	4	−	−	−	0.84	0.91
	5	−	−	−	−	0.85
202	1	0.84	0.24	0.67	0.83	0.28
	2	−	0.29	0.68	0.90	0.29
	3	−	−	0.17	0.27	0.52
	4	−	−	−	0.69	0.10
	5	−	−	−	−	0.25
203	1	0.25	0.29	0.29	0.18	0.39
	2	−	0.90	0.86	0.42	0.33
	3	−	−	0.91	0.50	0.36
	4	−	−	−	0.49	0.35
	5	−	−	−	−	0.30
204	1	0.84	0.78	0.81	0.72	0.83
	2	−	0.81	0.79	0.72	0.84
	3	−	−	0.81	0.83	0.80
	4	−	−	−	0.71	0.83
	5	−	−	−	−	0.74

Addressing such an issue, however, is beyond the scope of the current paper; more detailed features of the groups were not manipulated nor assessed in the study. Although low levels of dyadic synchrony would constrain the ability to have group synchrony, it is important to note that a group could have a high mean level of dyadic synchrony (e.g., ρ_*d*,group_ close to 1), without having high levels of group synchrony (e.g., ρ_group_ approximately 0.5), since they are measuring different processes. The distinction between group mean dyadic synchrony and group synchrony is that group synchronization measures the extent to which at any moment in time the interactions between all group members establish a “central” group behavior that acts in turn as an attractor for every individual member, whereas dyadic synchronization measures the degree to which two particular group members synchronize their behavior over the course of time when we considered them isolated from the remaining group members. This central group behavior reflects a mutuality and interdependence of influence. This attractor might not even be an observed state that an individual or a dyad is achieving—much as a prototype for a given cognitive category captures something about category members as a whole and might not be something ever specifically observed in any members (Rosch and Lloyd, 1978). Thus, measures of group level synchrony would be expected to relate to psychological and physical factors that lead to a strong sense of “groupness”—group entitativity or cohesiveness. The benefits of investigating these other levels of synchrony, individual, and dyadic, are that they allow us to empirically test the extent with which the synchrony process is a process that emerges as a result of relatively unidirectional influences.

## Conclusion

Here we presented and tested an analysis method proposed by Frank and Richardson (2010) for measuring the magnitude and patterning of the movement synchrony that can occur between the movements of a group of individuals. We experimentally tested the multivariate time-series method using a group-based rocking chair paradigm in which six participants positioned in a circle rocked in rocking chairs. In addition to instructing participants to intentionally coordinate their rocking chair movements with their eyes-open, we also instructed groups to rock in an eyes-closed condition in order to test whether the cluster phase statistics could effectively differentiate between intentional and chance levels of group synchrony. In particular we were interested in determining (1) whether the cluster phase statistic ρ_group_ could effectively measure the overall synchrony of a group of individuals as a whole and, (2) the degree to which ρ_*k*_ could effectively determine whether the movements of any one individual in the group are synchronized to the movements of a group as a whole.

The results revealed that ρ_group_ and ρ_*k*_ did provide effective measures of (1) and (2), respectively, in that both statistics could statistically differentiate between the intentional (eyes-open) and chance level (eyes-closed) conditions (see Figures 2 and 3). In addition, the results revealed that the cluster phase measures of the mean and SD of relative phase (i.e., ${\bar{\phi }}_{\text{clus}.}$ and SDϕ_clus._, respectively) can be used to identify the patterning of the synchrony that emerges. More specifically, the data presented in Table 1 demonstrates how ${\bar{\phi }}_{\text{clus}.}$ and SDϕ_clus._ can be used to determine whether individuals are coordinated to the group as a whole in an inphase (0°) or antiphase (180°) manner, or in some other stable relative-phase relation (e.g., 90°, 45°). Consistent with past research using rocking chairs (Richardson et al., 2007), relative phases near inphase were the dominant pattern.

We also illustrated how this analysis can be conducted within the context of considering all levels of influence (dyadic as well as group) within a group. Although we did not have any manipulations in this study that would lead dyadic processes to be crucial factors, we have illustrated how such analyses would be conducted. In situations where pairs of allies are present within a group of strangers or allies are absent, and in situations where one individual is a group leader versus when a leader is absent, the patterns of dyadic level synchrony should distinguish these different groups.

At present the cluster phase method presented here cannot account for coupling delays or leading/following behavior. However, it is plausible to assume that the method could be generalized to take such effects into account. First, the Kuramoto model with delay has been studied in the literature (e.g., Huber and Tsimring, 2003) and data analysis techniques have been developed to determine the underlying evolution equations of such stochastic delay systems (see e.g., Frank et al., 2004, 2005). The challenge in this context is that it is difficult to distinguish between the two kinds of couplings that lead to the same observation: a coupling without delay such that the attractor is at a particular phase difference different from zero and a coupling with delay e.g., with an in-phase attractor. Therefore, generalizing the current approach to account for delays is not a trivial matter. Mathematical models for leading/following behavior have been proposed for example for group and jury decision-making (e.g., Boster et al., 1991). It might be possible, therefore, for the Kuramoto model and cluster phase method presented here to be generalized in an analogous way and, thus, future work should be directed accordingly.

In the current study, we employed an eyes-closed condition as a control condition by providing a measure of chance level coordination. It is worth noting, however, that one could generate surrogate data for control purposes—i.e., by shuffled recorded data or by generating data with known random influences based on the recorded data (see e.g., Schreiber and Schmitz, 2000). Such surrogate data analysis would enable one to quantify chancel level group synchrony (as well as individual and dyadic synchrony) without the need for a control condition or control trials (i.e., no visual or non-coupled movement trials). For some experimental designs this may be preferable. For instance, when multiple trials or specific control trials may unduly influence participants' movements or may reveal the true nature of the study (i.e., when investigating spontaneous or unintentional coordination).

Researchers may have some concerns regarding whether these methods are constrained to situations where group members are seated, and seated in rocking chairs in particular. Although the analyses presented here all involve analysis of rhythmic (periodic) behavior, it is important to note that the analyses could be extended to situations where the movements involve natural gestural or postural movements during conversation (Schmidt et al., 2011) or movement during dance (Himberg and Thompson, 2010; Van Dyck et al., 2010). It is also possible that the analysis proposed could even be adapted to quantify group cognitive behavior and performance (e.g., Woolley et al., 2010).

By validating the cluster phase method proposed by Frank and Richardson (2010) the current study provides a practical demonstration of how researchers interested in group synchrony can objectively measure the magnitude of such synchrony. Accordingly, the cluster phase statistics could be used in future research to determine whether and how the magnitude and stability of group synchrony influences the social dynamics of group interaction. In particular, we would predict that strength of group synchrony is correlated with self (and perceiver) reports of group entitativity, cohesiveness, and identification with the group. More broadly, it seems likely that the cluster phase method will aid social scientists interested in investigating the dynamic time-dependent structure of group behavior, with respect not only to movement synchrony and group dynamics, but a broad spectrum of human perception and action phenomena.

## Author note

Example MATLAB code for the cluster phase method can be downloaded from http://homepages.uc.edu/~richamo/downloads.html. Example data can also be downloaded for demonstration purposes and for testing the analysis code.

### Conflict of interest statement

The authors declare that the research was conducted in the absence of any commercial or financial relationships that could be construed as a potential conflict of interest.

## Footnotes

^1^Classically, the Kuramoto method is defined in the thermodynamic limit (i.e., for systems composed of an extremely large number N of oscillatory units such that the limit N → ∞ can be considered as a good approximation; Kuramoto, 1984, 1989). Frank and Richardson, however, described how this method can be adapted to quantify phase synchronization in multivariate time series when the number N of movement time-series or trajectories is relatively small compared to the thermodynamic limit (i.e., *N* can be 5, 10, 25, 50, or 100).

^2^Circular variance is a circular or directional statistic that measures the spread of a set of dihedral angles. See Fisher (1993) for more details.

^3^Although not demonstrated here, ρ

^4^The participants and data presented here are not the same as reported by Frank and Richardson (2010).

^5^Extensive pilot testing revealed that individuals naturally rocked at between 0.5 and 0.7 Hz. Thus, 0.6 Hz was chosen as it represented the average self-selected (comfort mode) frequency of individuals.
